# A Comparative Randomized Controlled Clinical Trial on the Effectiveness, Safety, and Tolerability of a Homeopathic Medicinal Product in Children with Sleep Disorders and Restlessness

**DOI:** 10.1155/2016/9539030

**Published:** 2016-05-08

**Authors:** Miek C. Jong, Lydia Ilyenko, Irina Kholodova, Cynthia Verwer, Julia Burkart, Stephan Weber, Thomas Keller, Petra Klement

**Affiliations:** ^1^Department Nutrition & Health, Louis Bolk Institute, 3972 Driebergen, Netherlands; ^2^Department of Health Sciences, Mid Sweden University, 85170 Sundsvall, Sweden; ^3^National Information and Knowledge Center on Integrative Medicine (NIKIM), Amsterdam, Netherlands; ^4^Russian State Medical University, Moscow 117997, Russia; ^5^Deutsche Homöopathie-Union, DHU-Arzneimittel GmbH & Co. KG, 76227 Karlsruhe, Germany; ^6^Acomed statistik, 04275 Leipzig, Germany

## Abstract

A prospective, multicenter, randomized, open-label, controlled clinical trial was performed to evaluate the effectiveness and safety of the homeopathic product ZinCyp-3-02 in children with sleep disorders for ≥ one month compared to glycine. Children ≤ six years old received either ZinCyp-3-02 (*N* = 89) or comparator glycine (*N* = 90). After treatment for 28 days, total sleep-disorder-associated complaints severity scores decreased in both groups from median 7.0 (out of maximum 11.0) points to 2.0 (ZinCyp-3-02) and 4.0 (glycine) points, respectively, with overall higher odds of showing improvement for ZinCyp-3-02 (odds ratio: 4.45 (95% CI: 2.77–7.14), *p* < 0.0001,* POM overall treatment related effect*). Absence of individual complaints (time to sleep onset, difficulties maintaining sleep, sleep duration, troubled sleep (somniloquism), physical inactivity after awakening, restlessness for unknown reason, and sleep disorders frequency) at study end were significantly higher with ZinCyp-3-02 (all *p* values < 0.05). More children with ZinCyp-3-02 were totally free of complaints (*p* = 0.0258). Treatment effectiveness (*p* < 0.0001) and satisfaction assessments (*p* < 0.0001) were more favorable for ZinCyp-3-02. Few nonserious adverse drug reactions were reported (ZinCyp-3-02: *N* = 2, glycine: *N* = 1) and both treatments were well tolerated. Treatment with the homeopathic product ZinCyp-3-02 was found to be safe and superior to the comparator glycine in the treatment of sleep disorders in children.

## 1. Introduction

Sleep problems occur frequently in children, with a prevalence of approximately 30 to 50% [1, 2]. The most common types of sleep problems in children are difficulties falling asleep (e.g., bedtime problems) and difficulties maintaining sleep (e.g., night time waking) [2]. Inadequate sleep in children may have a negative impact on their cognitive development, mood regulation, attention, behavior, and quality of life [3]. Not only are children affected, but also parents and caregivers are affected in their wellbeing and daily working activities because of sleep deprivation [4]. Therefore, there is an urgent need to identify and treat sleep disorders in children. Current management strategies for sleep disorders start with educating parents about sleep hygiene and adequate sleep routines [5]. Other behavioral therapies, such as cognitive behavioral therapy, have also shown to improve sleep quality in young children [6]. When sleep hygiene and behavioral interventions fail to have an effect, pharmacologic treatment with, for example, antihistaminic agents, alpha-agonists, or benzodiazepines may be considered [7]. It should be emphasized that these drugs are often used off-label, as there exist no approved drugs for treating sleep disorders in children [2, 7]. But a randomized placebo-controlled trial demonstrated that the antihistaminic drug diphenhydramine was not more effective than placebo in the treatment of sleep disorders in infants [8]. Furthermore, these drugs should be prescribed with caution for children, as they are associated with a risk of side effects such as daytime sedation, dizziness, change in behavior, memory deficits, and paradoxical hyperactivity [2, 7]. Since pharmacologic treatment strategies for insomnia are limited, parents may seek other, natural products to meet the medical needs of their children.

It has been reported that complementary and alternative medicines (CAM), such as acupuncture, yoga, homeopathy, and traditional herbal products like chamomile and valerian, are used by people to treat sleep problems [9–14]. Homeopathic medications (single remedies or complex homeopathic products), one of the CAM treatment options for sleep problems, either are used in self-management (bought over the counter) or are prescribed by homeopathic practitioners [15]. Several placebo-controlled trials have investigated the efficacy of homeopathy in the treatment of adults with sleep problems (insomnia) with inconsistent results. In a systematic review including four randomized controlled trials (RCTs), no statistically significant differences were found between homeopathic and placebo treatment for insomnia [16]. Since then, one RCT on individualized homeopathy has been published that found a significant increase in the duration of sleep throughout the study in favor of homeopathy compared to placebo [17]. Another RCT with a complex homeopathic medicinal product reported that homeopathic treatment significantly increased sleep quality compared to placebo treatment [18]. Clearly, further studies are needed to establish the efficacy of homeopathy in the treatment of insomnia.

According to our knowledge, no RCTs have yet been published on the effects of homeopathy in the treatment of sleep problems in children. The current study was initiated to investigate the effectiveness, safety, and tolerability of ZinCyp-3-02, a homeopathic complex product that is sold over the counter in many European and non-European countries for the treatment of restlessness and sleep disorders in babies and children. Two previous single arm observational studies have shown that ZinCyp-3-02 may improve sleep disorders in children [19, 20]. In the present randomized study, the homeopathic product ZinCyp-3-02 was compared to glycine, a nonessential amino acid with shown efficacy in the treatment of insomnia [21–23]. The current comparative effectiveness study is in line with the clinical study designs as recommended by CAMbrella in their roadmap for future CAM research [24].

## 2. Material and Methods

### 2.1. Study Design

The study was designed as a prospective, multicenter, randomized, open-label, comparative, controlled clinical trial with two parallel groups to obtain clinical data for regulatory purposes as required for marketing authorization of ZinCyp-3-02 in the Russian Federation (RF). Comparing the effectiveness and safety of ZinCyp-3-02 with a comparator product (glycine) already marketed in the RF for treatment of sleeping disorders in children was requested by the Russian regulatory authorities. The study was conducted in five outpatient pediatric clinics in the RF in accordance with the legislation of the RF and its national standards of Good Clinical Practice. The five centers included the Children City Clinical Hospital No. 13 of the State Educational Institution for Higher Professional Education (Russian State Medical University), the Children City Policlinic No. 86 of the State Educational Institution for Additional Professional Education (Russian Medical Academy of Postgraduational Education), and the Federal State Institution (Moscow Scientific-Research Institute of Pediatrics and Children Surgery) in Moscow, the Children Regional Clinical Hospital 30V of the State Educational Institution for Higher Professional Education (Smolensk State Medical Academy) in Smolensk, and the State Educational Institution for Higher Professional Education (Yaroslavl State Medical Academy) in Yaroslavl, Russia.

The individual duration of time in the study was four weeks (28 days) for each child who participated in the study. No run-in or posttreatment period was included. A total of four study visits were scheduled including the baseline visit on day 0 (V1), a follow-up visit on days 3–5 (V2) and day 14 (V3), and the study termination visit on day 28 (V4), which also included the final overall evaluation.

### 2.2. Ethics and Regulatory Approvals

Prior to study initiation, the study protocol was approved by the independent ethics committee of the RF (Protocol number 66, May 12, 2010) and by the appropriate local ethics committees of the participating study centers, as well as by the regulatory authority of the Ministry of Health and Social Development of the RF (Protocol number 253, May 31, 2010). Approval for a one-year prolongation of the study was obtained by the Ministry of Health and Social Development of the RF (Permission number 273291-31-1, December 31, 2010). The data of the present clinical trial were first analyzed in RF and submitted to the Ministry of Health and Social Development of the RF in June 2011. The data presented in this publication are based on a new analysis from 2014 to 2015, in line with International Conference on Harmonization (ICH) guidelines.

### 2.3. Study Population

Children had to meet the following inclusion criteria in order to participate in the study: children who are of both genders, who are up to six years of age, and who have sleep disorders which manifested in difficulties falling asleep and maintaining sleep, present for at least one month prior to study start. Children were excluded from participation, if their sleep disorders were associated with somatic or psychic diseases and intracranial hypertension or in case of severe concomitant diseases (renal failure, heart anomalies, circulatory failure, cardiomyopathy, decompensated kidney and liver, immunosuppressive conditions, and oncological diseases), known or suspected hypersensitivities to the components of the study medications, participation in clinical studies on other medications within the past six months before the start of the study, and the use of any other medications with sedative, soporific, or psychostimulant action within the last 30 days prior to study start. During the study period children were not allowed to use these medications, either. Any other treatment during the course of the study was allowed, if it could not be discontinued for medical reasons. The age of the enrolled children was divided into three groups: children < one year of age, children between one and three years of age, and children ≥ four years of age. Inclusion was monitored in order to obtain a comparable amount of children in each age group. The first child was included in the study on September 9, 2010, and the last child completed the study on May 8, 2011.

### 2.4. Intervention

Children with sleep disorders were randomly assigned to the intervention group (ZinCyp-3-02) or the control group (glycine). Both groups received treatment for four weeks (28 days). The intervention group was treated with the investigational product ZinCyp-3-02 tablets with the dosage regimen of one tablet, four times a day. For children younger than three years of age, the ZinCyp-3-02 tablet had to be dissolved in 5 mL (one teaspoon) of water to facilitate administration. ZinCyp-3-02 (Dormikind®, Deutsche Homöopathie-Union, DHU-Arzneimittel GmbH & Co. KG) is a complex homeopathic medicinal product containing three active ingredients:* Cypripedium pubescens* D4,* Magnesium carbonicum* D10, and* Zincum valerianicum* D12. The control group was treated with the comparator medicinal product aminoacetic acid glycine tablets, containing 100 mg glycine per tablet (glycine, Biotics PMBCs, Ltd.). Based on the recommendations in the Russian package leaflet of glycine, the dosage regimen for children between three and six years old was one tablet, two times a day. For children younger than three years of age, half a tablet had to be taken two times a day for two weeks and thereafter half a tablet once a day. The half tablet had to be crushed and mixed with 5 mL (one teaspoon) of water to facilitate intake.

### 2.5. Randomization

The randomization list with a block size of four was generated by the Laboratory of Biostatistics State Research Center for Preventative Medicine (Moscow, RF). According to the randomization list, 50% of the children were allocated to the ZinCyp-3-02 group and 50% to the control group. For each child, the investigator at the respective center received a numbered, sealed, random envelope containing the information on the study medication to be given to the child. The envelope with the lowest available number was opened at the center after the child's parents had provided signed informed consent.

### 2.6. Outcome Parameters

The objective of the present study was to assess the effectiveness, safety, and tolerability of the homeopathic medicinal product ZinCyp-3-02 in children with sleep disorders and restlessness compared to the medicinal product glycine. Primary study outcome was the change in total complaints severity score (time to sleep onset, sleep duration, physical inactivity, and slowness of movements after awakening, each of the previous complaints with a maximum of one point; difficulties maintaining sleep, troubled sleep (somniloquism), restlessness for unknown reason, and sleep disorders frequency, each of them with a maximum of two points; maximum total score of eleven points) assessed by investigators according to parents' answers at each study visit (day 0 (V1), days 3–5 (V2), day 14 (V3), and day 28 (V4)). In order to assess the clinical relevance of observed changes for each study visit in primary outcome, two responsiveness criteria were defined: (a) absence of all complaints and (b) reduction in number of present complaints of at least 50% compared to baseline. Secondary outcome parameters were the severity of individual complaints assessed by investigator according to parents' answers at each study visit and overall treatment outcome assessed at each follow-up and termination visit as well as in the final overall evaluation by children/parents and investigator using the Integrative Medicine Outcome Scale (IMOS), a 5-point verbal rating scale (no complaints, major improvement, improvement, no change, and deterioration) [25]. Another secondary outcome parameter was satisfaction with treatment assessed by children/parents in the final overall evaluation at the termination visit using the Integrative Medicine Satisfaction Scale (IMPSS), a 5-point verbal rating scale (very satisfied, satisfied, undecided, dissatisfied, and very dissatisfied) [25]. Safety of the investigational products was evaluated by adverse events (AEs) monitoring. Assessment of treatment tolerability was evaluated by investigator as well as children/parents at follow-up and termination visits and in the final overall evaluation using a 4-point verbal rating scale (very good, good, satisfactory, and poor).

### 2.7. Sample Size

The study has an explorative character and therefore a formal sample size calculation was not necessarily required. The planned number of children was justified as follows: to detect a difference in effectiveness between the two investigational products, an effect of ZinCyp-3-02 in 90% of children with sleep disorders and an effect of the comparator product (control) in 70% of children with sleep disorders at study end were estimated, whereby the fraction of patients with a total complaints severity score ≤ 3 was used as measure of effectiveness. A sample size of 80 children in each group provided a power of 90% assuming a significance level of 0.05. This sample size can be regarded as a conservative estimate since in the final analysis an ordinal outcome was analyzed as primary endpoint instead of binary outcome.

### 2.8. Statistical Analyses

All analyses were based on the intention-to-treat (ITT) principle. The ITT population included those patients who entered into treatment, had received at least one dose of study medication, and had at least one postbaseline response measurement. Complete case analysis of ITT population was performed. Efforts regarding handling of missing values were not required, since overall less than 5% of information was missing. Homogeneity of the two treatment groups was assessed by regarding possible clinical relevance of group-specific differences in demographic data and other data obtained at baseline visit. Primary study outcome was presented by descriptive statistics and corresponding box-whisker plots. As primary analysis method, changes in total complaints severity scores were investigated by proportional odds model (POM) taking into account study specific situation of repeatedly measured outcome. As the total complaints severity score at baseline may be regarded as an important factor for the outcome after treatment (e.g., high baseline scores reflect a higher degree of initial suffering from symptoms), it was included in the model as a covariate. Differences between treatment groups were presented as odds ratio (OR) estimates along with their two-sided 95% confidence intervals (CI) and related *p* values. Further responsiveness analysis on primary outcome (absence of all complaints and reduction in number of present complaints of at least 50% compared to baseline) was performed by means of repeated measures logistic regression. The estimated treatment effect was presented in terms of OR along with its two-sided 95% CIs. All secondary outcome parameters were presented by descriptive statistics in counts and percentages. Changes in individual complaints severity were evaluated by means of calculating the respective item's categories' proportion for absence and presence at each visit in relation to the baseline visit. To test treatment related differences for all secondary outcome parameters, Chi-square (*χ* ^2^) tests were performed. A rejection criterion of 0.05 was set for all statistical tests. If tests allowed, the statistics were two-tailed. SAS program version 9.2 was used for the statistical analysis.

## 3. Results

### 3.1. Patient Population

**Figure 1:**
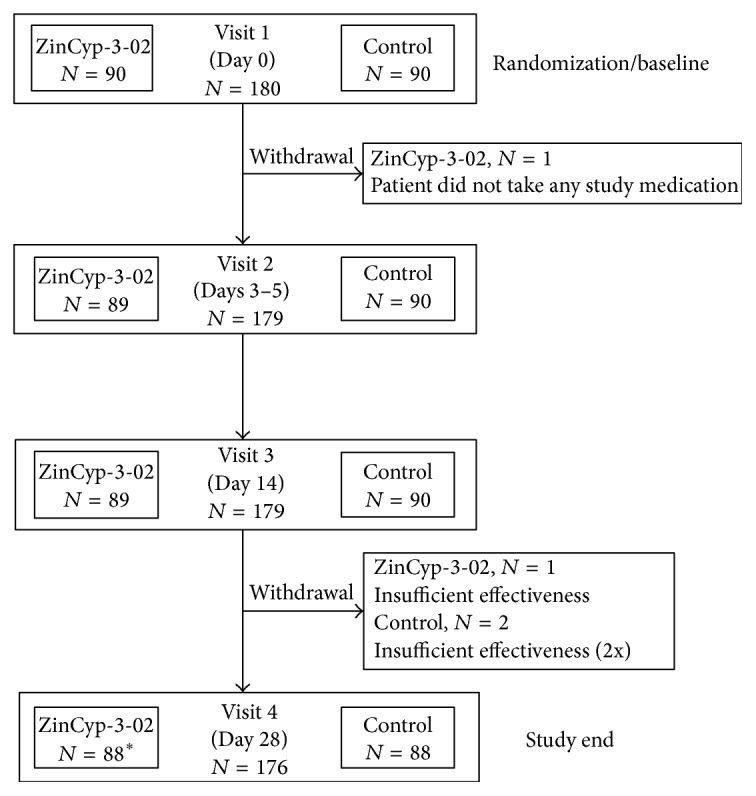
Flow diagram of children in the study.  ^*∗*^In addition to the three children who withdrew between Visit 3 and Visit 4, there was one child in the ZinCyp-3-02 group who completed the assessments for Visit 4 but who withdrew prematurely between Visit 3 and Visit 4 because of an adverse drug reaction. Intention-to-treat population consisted of 179 children, since one child in the ZinCyp-3-02 group did not take study medication.

In total, 180 children were included in the study of which 90 were allocated to the ZinCyp-3-02 group and 90 to the control group (Figure 1). One child in the ZinCyp-3-02 group did not take any study medication and terminated the study prematurely. Therefore, the ITT analysis included 179 children. Four other children terminated the study prematurely, two in the ZinCyp-3-02 group and two in the control group (Figure 1).  Table 1 summarizes demographic data and clinical characteristics of children in the study. Overall, there were no relevant differences between the treatment groups at baseline. Concomitant disease(s) (ZinCyp-3-02: 29.2% out of 89 children, control: 27.8% out of 90 children) and use of one or more concomitant medications (ZinCyp-3-02: 13.5% out of 89 children, control: 12.2% out of 90 children) were comparable between both treatment groups at baseline.

### 3.2. Primary Outcome

**Figure 2:**
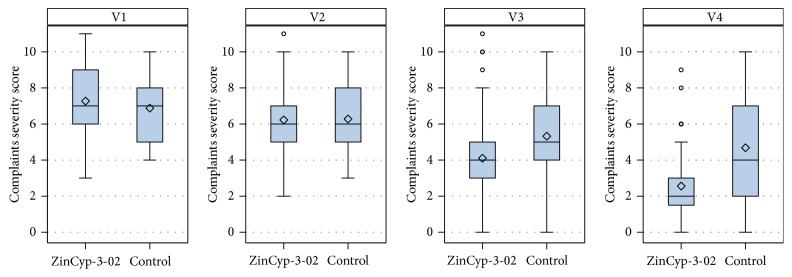
Primary outcome: change in total complaints severity score. Box-whisker plots showing mean, standard deviation, median, minimum, P25%, P75%, and maximum. V1 (baseline, day 0), V2 (days 3–5), V3 (day 14), and V4 (day 28). Intention-to-treat analysis.

Figure 2 shows the change in total complaints severity score as primary outcome. In the ZinCyp-3-02 group, the total complaints severity score decreased from median 7.0 points at baseline to 2.0 points at day 28 of treatment (V4). A decrease in the total complaints severity score was also observed in the control group but less pronounced at day 28 (median 7.0 points at baseline to 4.0 points at V4). The statistical evaluation of treatment related differences in total complaints severity score is shown in Table 2. For the overall treatment related difference an OR of 4.45 (95% CI: 2.77–7.14) was found, indicating that children in the ZinCyp-3-02 group had higher odds of showing improvement (as indicated by lower total complaints severity scores) than those in the control group (baseline adjusted POM: *p* < 0.0001; ITT). Similar significant differences in favor of ZinCyp-3-02 were found at day 14 (V3) and day 28 (V4) of treatment (Table 2). Responsiveness analysis found that a higher percentage of children in the ZinCyp-3-02 group had no complaints at study end (ZinCyp-3-02: 15.9% out of 88 children (data of withdrawn children were not replaced), control: 5.7% out of 88 children (data of withdrawn children were not replaced)). The calculated OR of 3.46 (95% CI: 1.16–10.31) thus demonstrated that children in the ZinCyp-3-02 group had higher odds of showing responsiveness defined as “absence of all complaints” than those in the control group (baseline adjusted simple logistic regression model: *p* = 0.0258; ITT). With respect to the other responsiveness criterion, reduction in complaints by at least 50%, a significant higher percentage of responders was found in the ZinCyp-3-02 group compared to the control group at study end (ZinCyp-3-02: 63.6% out of 88 children (data from withdrawn children were not replaced), control: 30.7% out of 88 children (data from withdrawn children were not replaced)). The calculated OR of 2.40 (95% CI: 1.14–5.07) indicated that the children in the ZinCyp-3-02 group had overall higher odds of showing responsiveness defined as “reduction in number of present complaints of at least 50% compared to baseline,” than those in the control group (baseline adjusted repeated logistic regression: *p* = 0.0217; ITT).

### 3.3. Secondary Outcome

Changes in individual complaints severity were evaluated as secondary outcome. As shown in Table 3, no significant differences in proportions of individual complaints' absence were found at baseline (day 0) and days 3–5 of treatment between the two treatment groups. At day 14 of treatment, a significant difference in proportion in favor of ZinCyp-3-02 was found for the individual complaints “time to sleep onset” (absence in ZinCyp-3-02 group: 65.2% (58 out of 89 children, of whom 13 did not have the complaint at baseline), absence in control group: 40.0% (36 out of 90 children, of whom 16 did not have the complaint at baseline)), “troubled sleep, somniloquism” (absence in ZinCyp-3-02 group: 37.1% (33 out of 89 children, of whom 12 did not have the complaint at baseline), absence in control group: 23.3% (21 out of 90 children, of whom nine did not have the complaint at baseline)), and “sleep disorders frequency” (absence in ZinCyp-3-02 group: 9.0% (8 out of 89 children), absence in control group: 1.1% (1 out of 90 children)), indicating that these individual complaints were significantly more absent than present in the ZinCyp-3-02 group compared to the control group. At study end (day 28) significant differences in favor of ZinCyp-3-02 were found with respect to absence/presence proportion for all individual complaints (Table 3).

Another secondary outcome variable was the evaluation of the effectiveness of ZinCyp-3-02 tablets and comparator tablets (control group), assessed by the investigator and children/parents in the study by means of the IMOS. As shown in Table 4, in the final overall evaluation at study end (day 28) the highest values for major improvement were assessed in the ZinCyp-3-02 group (investigator: 44.9%, children/parents: 39.3%), whereas the peak value in the control group was assessed for no change (investigator: 46.7%, children/parents: 43.3%). On average, one-fifth (investigator: 15.7%, children/parents: 22.5%) of the children in the ZinCyp-3-02 group appeared to have no complaints at the end of the study, whereas only a small percentage (investigator: 5.6%, children/parents: 6.7%) in the control group reported no complaints (Table 4). The differences in IMOS classification frequencies as observed between the ZinCyp-3-02 and control group were found to be significantly different (Table 4). At all other earlier visits and at termination visit, treatment effectiveness assessment showed significant differences between the treatment groups in favor of ZinCyp-3-02 (results not shown).

**Figure 3:**
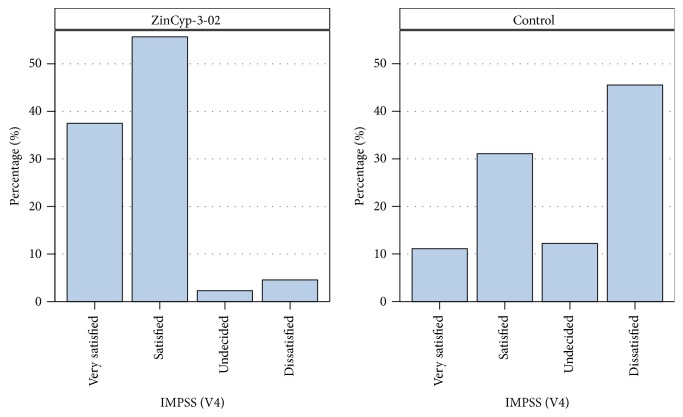
Treatment satisfaction assessments by children/parents by means of IMPSS. IMPSS: Integrative Medicine Patient Satisfaction Scale, assessment at study end (V4, day 28, final overall evaluation). Intention-to-treat analysis.

Treatment satisfaction with ZinCyp-3-02 tablets and comparator tablets was assessed by children/parents at study end (at termination visit in the final overall evaluation) by means of the IMPSS (Figure 3). Most children/parents were either satisfied (55.1% out of 89 children) or very satisfied (37.1% out of 89 children) with ZinCyp-3-02 treatment results. In the control group, more children/parents were dissatisfied (45.6% out of 90 children) with the treatment compared to those satisfied (31.1% out of 90 children) or very satisfied (11.1% out of 90 children) (Figure 3). These differences were found to be significant (*χ* ^2^-test: *p* < 0.0001; ITT) between the treatment groups, with higher treatment satisfaction ratings for ZinCyp-3-02 tablets.

### 3.4. Safety and Tolerability

Overall, eleven AEs were reported, six in the ZinCyp-3-02 and five in the control group. All AEs were nonserious and mild or moderate in intensity. The most common AEs reported were acute upper respiratory tract infections (ZinCyp-3-02: *N* = 4, Control: *N* = 2). Other AEs reported in the ZinCyp-3-02 group were excitability (*N* = 1) and nervousness (*N* = 1). In the control group, otalgia (*N* = 1), food allergy (*N* = 1), and excitement (*N* = 1) were reported. Nervousness was evaluated as having a possible causal relationship to the treatment with ZinCyp-3-02 tablets. Excitability was considered to be unlikely/improbable related to ZinCyp-3-02 tablets, whereas the remaining four AEs in the ZinCyp-3-02 group were considered not to be related to ZinCyp-3-02 intake. Excitement as reported in the control group was classified by the investigator as having a probable causal relationship to treatment with glycine tablets and the other four AEs were assessed as not related to treatment. One child in the ZinCyp-3-02 group withdrew from the study because of the occurrence of one adverse drug reaction (excitability).

As shown in Table 5, the majority of investigators and children/parents rated the tolerability of both treatments as “very good” (ZinCyp-3-02: 62-63 out of 89 children (69.7–70.8%), control: 58-59 out of 90 children (64.4–65.6%)) or “good” (ZinCyp-3-02: 25-26 out of 89 children (28.1–29.2%), control: 25-26 out of 90 children (27.8–28.9%)).

## 4. Discussion

According to our knowledge the present study is the first RCT that reports on the effectiveness of a homeopathic intervention in the treatment of sleep disorders in children. A four-week treatment with the homeopathic product ZinCyp-3-02 tablets was found to be more effective in reducing sleep-disorder associated complaints than the comparator product glycine that is approved in RF for treating sleep disorders in children. Complaints related to sleep quality, as well as the frequency of sleep disorders and time to sleep onset significantly improved more often under treatment with ZinCyp-3-02 tablets compared to glycine tablets. The clinical relevance of the observed superiority of ZinCyp-3-02 tablets over glycine was confirmed by the findings that significantly more children in the ZinCyp-3-02 group were either free of complaints or exhibited a 50% or larger reduction in complaints severity scores. ZinCyp-3-02 tablets also scored better than comparator tablets with respect to absence of the complaint “restlessness for unknown reason” and effectiveness assessments by investigator and children/parents at study end. These significant differences in favor of ZinCyp-3-02 tablets were for most outcome parameters already apparent after two weeks of treatment. Glycine was evaluated as a suitable comparator to investigate the comparative effectiveness of ZinCyp-3-02 tablets, since previous studies have demonstrated that glycine significantly improves sleep quality compared to placebo in healthy volunteers who were dissatisfied with sleep quality [22, 23] and in partly sleep-restricted healthy volunteers [21].

Treatment with ZinCyp-3-02 tablets appeared to be safe and well tolerated and almost all children and parents (92.1% out of 89 children) were “satisfied/very satisfied” with it. These findings are in line with other studies confirming that homeopathic medicines are well tolerated in children in general and that adverse drug reactions are very rare [26–29]. In contrast, the use of pharmaceutical agents such as antihistamines, alpha-agonists, or benzodiazepines may pose safety risks to children with insomnia. Antihistamines and benzodiazepines are not approved to treat insomnia in children [2] and may be associated with developing a tolerance (necessitating increasing doses), a risk of habituation or addiction [30], and other serious side effects in children [2, 4, 7]. In rare cases, fatal intoxication has been reported with the use of diphenhydramine, an antihistamine, in children [31]. Altogether, these findings suggest that the homeopathic product ZinCyp-3-02 is an interesting and safe treatment alternative for sleeping disorders in children compared to pharmacological treatment.

Several mechanisms have been proposed by which the comparator in this study, glycine, can influence physiological sleep regulation. Studies in rats and mice demonstrated that glycine may affect sleep through regulation of neuropeptides in suprachiasmatic nucleus [21], by enhancement of extracellular serotonin in the prefrontal cortex [32], or by inhibition of orexin neurons [33]. The underlying mechanisms by which the homeopathic ZinCyp-3-02 tablets regulate sleep are unknown and were not investigated in the present study. Several studies have reported that homeopathic medications can modulate sleep physiology in animal (rats and mice) models [34–38]. By means of electroencephalography, it was demonstrated that the homeopathic medications* Coffea cruda* and* Nux vomica* significantly alter sleep patterns and sleep intensity compared to placebo [34–36, 38]. Further studies are necessary to investigate whether ZinCyp-3-02 tablets may exert similar effects on sleep regulation.

The observed findings in the present study should be regarded in the light of its limitations. The unblinded study design might have impacted the subjective assessment of sleep-associated complaints and other outcomes, partly explaining the observed superiority of ZinCyp-3-02 tablets over glycine tablets. Another limitation of the present study is that the chosen outcome variables were subjective assessments of either investigators or investigators and children/parents using nonvalidated scales. The use of objective markers, such as polysomnography, seems to be successfully applied in homeopathic research. An experimental study with polysomnography (PSG) found that homeopathic medications increased total sleep time and nonrapid eye movement (NREM) sleep in young adults with a history of coffee-related insomnia [39]. Actigraphy has also been used as an objective marker to study the effect of particular interventions on sleep disorders in children [40]. Actigraphy can be applied in the form of wrist watches. It monitors body movement of children to evaluate their sleep-wake rhythm. Future studies with such objective measurements as PSG and actigraphy are recommended to further examine the effects of ZinCyp-3-02 on sleep quality and quantity in children. Another limitation of this study was that although glycine was authorized in the RF for the treatment of pediatric sleeping disorders, the efficacy of glycine has not been studied until now in this specific young-age group of children. Despite these limitations, the current study involved a large number of children with few dropouts and was of a randomized comparative design, increasing the precision of the study. Furthermore, the chosen study design closely resembled everyday clinical practice where diagnosis and treatment are also guided by subjective monitoring of complaints associated with sleep disorders such as bedtime resistance and difficulties falling asleep [41].

Strategies to treat sleep problems in children are of utmost importance, since sleep deprivation has a large effect on their cognitive performance [42] and other aspects related to behavioral and quality of life [3]. Until now, behavioral treatment strategies for global management of sleeping disorders have shown to be most effective in children [5, 6]. A review of 52 studies regarding behavioral treatments for bedtime problems and night awakenings demonstrated that a clinically relevant improvement was observed in at least 80% of treated children with insomnia [43]. Integration of treatment with the homeopathic product ZinCyp-3-02 in these behavioral interventions is proposed to maximize its effectiveness.

## 5. Conclusions

The homeopathic product, ZinCyp-3-02 tablets, was found to be superior to the comparator glycine in the reduction of complaints related to sleep quality, frequency of sleep disorders, time to sleep onset, and restlessness in children. Treatment with ZinCyp-3-02 tablets was safe and very well tolerated. Future studies with objective outcomes are warranted to further investigate the effects of ZinCyp-3-02 tablets on sleep regulation in children.

## Figures and Tables

**Table 1:** Demographic and clinical characteristics.

Demographic data		ZinCyp-3-02 group	Control group
Age groups (*N*)	<1 year	30	32
	1–3 years	36	35
	≥4 years	23	23

Boys/girls (*N*)	<1 year	19/11	18/14
	1–3 years	23/13	20/15
	≥4 years	10/13	13/10

Age in months (mean ± SD (*N*))	All age groups	30.1 ± 23.0 (89)	28.7 ± 23.2 (90)

Clinical characteristics		Mean ± SD (*N*)	Mean ± SD (*N*)

Duration of sleep disorders in months by time of inclusion	<1 year	2.2 ± 1.0 (29)	2.2 ± 1.0 (32)
	1–3 years	7.8 ± 6.2 (35)	8.0 ± 9.2 (35)
	≥4 years	8.2 ± 7.2 (23)	7.7 ± 11.7 (22)

SD: standard deviation. Intention-to-treat analysis.

**Table 2:** Odds ratios for primary outcome: change in total complaints severity score.

Visit	Odds ratio (OR) (ZinCyp-3-02 versus control)			*p* value
	Estimate	Lower bound 95% CI	Upper bound 95% CI	
V2 (days 3–5)	1.31	0.88	1.95	0.1879
V3 (day 14)	5.79	3.23	10.41	**<0.0001**
V4 (day 28)	11.64	5.92	22.88	**<0.0001**
Overall	4.45	2.77	7.14	**<0.0001**

OR: odds ratio (i.e., estimated odds of getting lower total complaints severity score in children treated with ZinCyp-3-02 divided by the estimated odds of getting lower total complaints severity score in children treated with the control as obtained from proportional odds model); CI: confidence interval. Intention-to-treat analysis.

**Table 3:** Treatment related difference of absence vs. presence evaluation of individual complaints.

Individual complaints	Visit	Absence of complaints				*χ* ^2^-test	Estimated difference in proportion of absence (Δ_ZinCyp3-02−control_)		
		ZinCyp-3-02 *N* = 89^A^		Control *N* = 90^B^			Estimate (%)	Lower CL	Upper CL
		*N*	%	*N*	%				
Time to sleep onset	V1 (day 0)	13	14.6	16	17.8	*—*	*—*	*—*	*—*
	V2 (days 3–5)	19	21.4	25	27.8	*p* = 0.3178	−6.43	−19.00	6.14
	V3 (day 14)	58	65.2	36	40.0	**p** = 0.0007	25.17	11.01	39.33
	V4 (day 28)	74	84.1	46	52.3	**p** < 0.0001	31.82	18.88	44.75

Difficulties maintaining sleep	V1 (day 0)	11	12.4	8	8.9	*—*	*—*	*—*	*—*
	V2 (days 3–5)	19	21.4	11	12.2	*p* = 0.1022	9.13	−1.75	20.00
	V3 (day 14)	36	40.5	24	26.7	*p* = 0.0508	13.78	0.09	27.47
	V4 (day 28)	58	65.9	37	42.1	**p** = 0.0015	23.86	9.56	38.16

Sleep duration (per day)	V1 (day 0)	34	38.2	43	47.8	*—*	*—*	*—*	*—*
	V2 (days 3–5)	46	51.7	50	55.6	*p* = 0.6037	−3.87	−18.47	10.73
	V3 (day 14)	63	70.8	56	62.2	*p* = 0.2249	8.56	−5.20	22.33
	V4 (day 28)	75	85.2	56	63.6	**p** = 0.0010	21.59	9.10	34.08

Troubled sleep (somniloquism)	V1 (day 0)	12	13.5	9	10.0	*—*	*—*	*—*	*—*
	V2 (days 3–5)	20	22.5	14	15.6	*p* = 0.2382	6.92	−4.54	18.37
	V3 (day 14)	33	37.1	21	23.3	**p** = 0.0451	13.75	0.44	27.05
	V4 (day 28)	46	52.3	30	34.1	**p** = 0.0149	18.18	3.79	32.57

Physical inactivity and slowness of movements after awakenings	V1 (day 0)	60	67.4	61	67.8	*—*	*—*	*—*	*—*
	V2 (days 3–5)	62	69.7	60	66.7	*p* = 0.6670	3.00	−10.64	16.64
	V3 (day 14)	71	79.8	63	70.0	*p* = 0.1317	9.78	−2.85	22.40
	V4 (day 28)	77	87.5	66	75.0	**p** = 0.0336	12.50	1.12	23.88

Restlessness for unknown reason	V1 (day 0)	23	25.8	30	33.3	*—*	*—*	*—*	*—*
	V2 (days 3–5)	33	37.1	31	34.4	*p* = 0.7131	2.63	−11.40	16.67
	V3 (day 14)	44	49.4	41	45.6	*p* = 0.6030	3.88	−10.74	18.50
	V4 (day 28)	57	64.8	44	50.0	**p** = 0.0475	14.77	0.32	29.22

Sleep disorders frequency	V1 (day 0)	0	0.0	0	0.0	*—*	*—*	*—*	*—*
	V2 (days 3–5)	2	2.3	1	1.1	*p* = 0.5538	1.14	−2.63	4.90
	V3 (day 14)	8	9.0	1	1.1	**p** = 0.0159	7.88	1.55	14.20
	V4 (day 28)	22	25.0	6	6.8	**p** = 0.0010	18.18	7.71	28.65

A: data for the one withdrawn child was not replaced for V4 (day 28), B: data for the two withdrawn children were not replaced for V4 (day 28), CL: confidence limit, and *χ* ^2^: Chi-square. Intention-to-treat analysis.

**Table 4:** Effectiveness assessments by investigators and children/parents by means of IMOS.

Assessment of IMOS	Investigator's assessment				*χ* ^2^-test	Children's/parents' assessment				*χ* ^2^-test
	ZinCyp-3-02 *N* = 89		Control *N* = 90			ZinCyp-3-02 *N* = 89		Control *N* = 90		
	*N*	%	*N*	%		*N*	%	*N*	%	
No complaints	14	15.7	5	5.6	**p** < 0.0001	20	22.5	6	6.7	**p** < 0.0001
Major improvement	40	44.9	17	18.9		35	39.3	16	17.8	
Improvement	31	34.8	26	28.9		30	33.7	29	32.2	
No change	3	3.4	42	46.7		3	3.4	39	43.3	
Deterioration	1	1.1	0	0.0		1	1.1	0	0.0	

Measurements at V4 (day 28) in the final overall evaluation. IMOS: Integrative Medicine Outcome Scale; *χ* ^2^: Chi-square. Intention-to-treat analysis.

**Table 5:** Tolerability assessment by investigator and children/parents.

Assessment of tolerability	Investigator's assessment				Children's/parents' assessment			
	ZinCyp-3-02 *N* = 89		Control *N* = 90		ZinCyp-3-02 *N* = 89		Control *N* = 90	
	*N*	%	*N*	%	*N*	%	*N*	%
Very good	63	70.8	59	65.6	62	69.7	58	64.4
Good	25	28.1	25	27.8	26	29.2	26	28.9
Satisfactory	0	0.0	6	6.7	0	0.0	6	6.7
Poor	1	1.1	0	0.0	1	1.1	0	0.0

Measurements at V4 (day 28) in the final overall evaluation. Intention-to-treat analysis.

## References

[1] Carter K. A., Hathaway N. E., Lettieri C. F. (2014). Common sleep disorders in children. *American Family Physician*.

[2] Nunes M. L., Bruni O. (2015). Insomnia in childhood and adolescence: clinical aspects, diagnosis, and therapeutic approach. *Journal of Pediatrics*.

[3] Beebe D. W. (2011). Cognitive, behavioral, and functional consequences of inadequate sleep in children and adolescents. *Pediatric Clinics of North America*.

[4] Owens J. A., Mindell J. A. (2011). Pediatric insomnia. *Pediatric Clinics of North America*.

[5] Allen S. L., Howlett M. D., Coulombe J. A., Corkum P. V. (2016). ABCs of SLEEPING: a review of the evidence behind pediatric sleep practice recommendations. *Sleep Medicine Reviews*.

[6] Meltzer L. J., Mindell J. A. (2014). Systematic review and meta-analysis of behavioral interventions for pediatric insomnia. *Journal of Pediatric Psychology*.

[7] Pelayo R., Yuen K. (2012). Pediatric sleep pharmacology. *Child and Adolescent Psychiatric Clinics of North America*.

[8] Merenstein D., Diener-West M., Halbower A. C., Krist A., Rubin H. R. (2006). The trial of infant response to diphenhydramine: the TIRED study—a randomized, controlled, patient-oriented trial. *Archives of Pediatrics and Adolescent Medicine*.

[9] Ekor M., Adeyemi O. S., Otuechere C. A. (2013). Management of anxiety and sleep disorders: role of complementary and alternative medicine and challenges of integration with conventional orthodox care. *Chinese Journal of Integrative Medicine*.

[10] Frass M., Strassl R. P., Friehs H., Müllner M., Kundi M., Kaye A. D. (2012). Use and acceptance of complementary and alternative medicine among the general population and medical personnel: a systematic review. *The Ochsner Journal*.

[11] Grimaldi-Bensouda L., Engel P., Massol J. (2012). Who seeks primary care for sleep, anxiety and depressive disorders from physicians prescribing homeopathic and other complementary medicine? Results from the EPI3 population survey. *BMJ Open*.

[12] Henry D., Rosenthal L., Dedrick D., Taylor D. (2013). Understanding patient responses to insomnia. *Behavioral Sleep Medicine*.

[13] Sánchez-Ortuño M. M., Bélanger L., Ivers H., LeBlanc M., Morin C. M. (2009). The use of natural products for sleep: a common practice?. *Sleep Medicine*.

[14] Sarris J., Byrne G. J. (2011). A systematic review of insomnia and complementary medicine. *Sleep Medicine Reviews*.

[15] Erlewyn-Lajeunesse M. (2012). Homeopathic medicines for children. *Archives of Disease in Childhood*.

[16] Cooper K. L., Relton C. (2010). Homeopathy for insomnia: a systematic review of research evidence. *Sleep Medicine Reviews*.

[17] Naudé D. F., Stephanie Couchman I. M., Maharaj A. (2010). Chronic primary insomnia: efficacy of homeopathic simillimum. *Homeopathy*.

[18] Hellhammer J., Schubert M. (2013). Effects of a homeopathic combination remedy on the acute stress response, well-being, and sleep: a double-blind, randomized clinical trial. *Journal of Alternative and Complementary Medicine*.

[19] Savoo V., Filonova T., Babenko E. (2012). Correcting sleep disturbances in young children. *Dytyachyy Likar*.

[20] Sukhonosova O., Molochek N. (2009). Experience of correcting symptomatology of increased excitability and sleep disturbances in children of early age. *Pediatriya, Akusherstvo ta Ginekologiya*.

[21] Bannai M., Kawai N., Ono K., Nakahara K., Murakami N. (2012). The effects of glycine on subjective daytime performance in partially sleep-restricted healthy volunteers. *Frontiers in Neurology*.

[22] Inagawa K., Hiraoka T., Kohda T., Yamadera W., Takahashi M. (2006). Subjective effects of glycine ingestion before bedtime on sleep quality. *Sleep and Biological Rhythms*.

[23] Yamadera W., Inagawa K., Chiba S., Bannai M., Takahashi M., Nakayama K. (2007). Glycine ingestion improves subjective sleep quality in human volunteers, correlating with polysomnographic changes. *Sleep and Biological Rhythms*.

[24] Fischer F. H., Lewith G., Witt C. M. (2014). High prevalence but limited evidence in complementary and alternative medicine: guidelines for future research. *BMC Complementary and Alternative Medicine*.

[25] Steinsbekk A. (1999). Data collection in homeopathic practice—a proposal for an international standard. *Homint R&D Newsletter*.

[26] Endrizzi C., Rossi E., Crudeli L., Garibaldi D. (2005). Harm in homeopathy: aggravations, adverse drug events or medication errors?. *Homeopathy*.

[27] Haidvogl M., Riley D. S., Heger M. (2007). Homeopathic and conventional treatment for acute respiratory and ear complaints: a comparative study on outcome in the primary care setting. *BMC Complementary and Alternative Medicine*.

[28] Jong M. C., Verwer C., van de Vijver L., Klement P., Burkart J., Baars E. (2015). Safety and tolerability of a homeopathic medicinal product for the treatment of painful teething in children. *Alternative & Integrative Medicine*.

[29] Michalsen A., Uehleke B., Stange R. (2015). Safety and compliance of a complex homeopathic drug (Contramutan N Saft) in the treatment of acute respiratory tract infections: a large observational (non-interventional) study in children and adults focussing on homeopathy specific adverse reactions versus adverse drug reactions. *Regulatory Toxicology and Pharmacology*.

[30] Shibley H. L., Malcolm R. J., Veatch L. M. (2008). Adolescents with insomnia and substance abuse: consequences and comorbidities. *Journal of Psychiatric Practice*.

[31] Baker A. M., Johnson D. G., Levisky J. A. (2003). Fatal diphenhydramine intoxication in infants. *Journal of Forensic Sciences*.

[32] Bannai M., Kawai N., Nagao K., Nakano S., Matsuzawa D., Shimizu E. (2011). Oral administration of glycine increases extracellular serotonin but not dopamine in the prefrontal cortex of rats. *Psychiatry and Clinical Neurosciences*.

[33] Hondo M., Furutani N., Yamasaki M., Watanabe M., Sakurai T. (2011). Orexin neurons receive glycinergic innervations. *PLoS ONE*.

[34] Ruiz-Vega G., Pérez-Ordaz L., Cortés-Galván L., Juárez-G F. M. (2003). A kinetic approach to caffeine—*Coffea cruda* interaction. *Homeopathy*.

[35] Ruiz-Vega G., Pérez-Ordaz L., León-Huéramo O., Cruz-Vázquez E., Sánchez-Diaz N. (2002). Comparative effect of Coffea cruda potencies on rats. *Homeopathy*.

[36] Ruiz-Vega G., Perez-Ordaz L., Proa-Flores P., Aguilar-Diaz Y. (2000). An evaluation of *Coffea cruda* effect on rats. *British Homeopathic Journal*.

[37] Ruiz-Vega G., Poitevin B., Pérez-Ordaz L. (2005). Histamine at high dilution reduces spectral density in delta band in sleeping rats. *Homeopathy*.

[38] Sukul A., Sinhabau S. P., Sukul N. C. (1999). Reduction of alcohol induced sleep time in albino mice by potentized Nux vomica prepared with 90% ethanol. *British Homeopathic Journal*.

[39] Bell I. R., Howerter A., Jackson N., Aickin M., Baldwin C. M., Bootzin R. R. (2011). Effects of homeopathic medicines on polysomnographic sleep of young adults with histories of coffee-related insomnia. *Sleep Medicine*.

[40] Cortesi F., Giannotti F., Sebastiani T., Panunzi S., Valente D. (2012). Controlled-release melatonin, singly and combined with cognitive behavioural therapy, for persistent insomnia in children with autism spectrum disorders: a randomized placebo-controlled trial. *Journal of Sleep Research*.

[41] Honaker S. M., Meltzer L. J. (2016). Sleep in pediatric primary care: a review of the literature. *Sleep Medicine Reviews*.

[42] Kelly Y., Kelly J., Sacker A. (2013). Time for bed: Associations with cognitive performance in 7-year-old children: a longitudinal population-based study. *Journal of Epidemiology and Community Health*.

[43] Mindell J. A., Kuhn B., Lewin D. S., Meltzer L. J., Sadeh A. (2006). Behavioral treatment of bedtime problems and night wakings in infants and young children. *Sleep*.

